# The Importance of Corporate Reputation for Sustainable Supply Chains: A Systematic Literature Review, Bibliometric Mapping, and Research Agenda

**DOI:** 10.1007/s10551-022-05268-x

**Published:** 2022-10-13

**Authors:** David von Berlepsch, Fred Lemke, Matthew Gorton

**Affiliations:** 1 Department of Marketing, Innovation and Organisation, Ghent University, Tweekerkenstraat 2, 9000 Ghent, Belgium; 2 Department of Marketing, Vlerick Business School, Bolwerklaan 21 bus 32, 1210 Brussels, Belgium; 3 Department of Marketing, Newcastle University Business School, 5 Barrack Road, Newcastle upon Tyne, NE1 4SE UK; 4 Department of Marketing, Ghent University, Tweekerkenstraat 2, 9000 Ghent, Belgium

**Keywords:** Corporate reputation, Supply chain, Literature review, Bibliometric mapping

## Abstract

Corporate Reputation (CR) is essential to value generation and is co-created between a company and its stakeholders, including supply chain actors. Consequently, CR is a critical and valuable resource that should be managed carefully along supply chains. However, the current CR literature is fragmented, and a general definition of CR is elusive. Besides, the academic CR debate largely lacks a supply chain perspective. This is not surprising, as it is very difficult to collect reliable data along supply chains. When supply chains span the globe, data collection is especially challenging, as the chain consists of multiple suppliers and subcontractors, positioned at different tier levels. Recognizing this, the paper examines firstly the current state of CR research through a systematic literature review from a business perspective. The review is combined with a bibliometric mapping approach to show the most influential research clusters, representative of CR research streams and their contributors. This process highlights that the connection between CR and supply chain issues represents a major research gap. Consequently, this paper introduces a research agenda connecting these the two traditionally separated research fields.

## Introduction

Corporate Reputation (CR) is an intangible and critical asset in sustaining business operations. Despite many years of efforts and initiatives in the private sector, politics, and academic research, addressing the importance of reputation, reputational risks, and reputation management along entire supply chains, the management of CR has not yet become established as an important part of strategic management decisions in practice. Although the many advantages that a positive CR brings to individual organizations (improving the bottom line, being a decisive factor in some customers’ choices, buffering for risks, etc.), CR cannot be seen in isolation (Dhingra & Krishnan, 2021). The shaping of stakeholders’ CR perceptions occurs through the interaction of stakeholders, especially business partners that make up the supply chain (Mani & Gunasekaran, 2021; Nguyen & Phan, 2021) Thus, a corporation’s overall reputation is influenced by the actions and behavior of its supply chain partners (Saleheen & Habib, 2022). Over time, however, a better understanding of the important role CR plays in the successful and sustainable development of companies emerged. Consequently, the CR topic has been discussed via a strategic management lens, over the past two decades. However, supply chains represent a nascent topic in CR debates, given that CR is an important dimension of supplier relationships with wider implications in chain settings (Fan et al., 2021).

This paper addresses two major research gaps regarding the interplay between supply chain management (SCM) and CR research. The first gap originates from the traditionally separated fields of CR and supply chain research, which have been treated as two different units of analysis, often in isolation and without understanding the linkages between them (Blom & Niemann, 2022). The second gap concerns the absence of a research agenda connecting these two fields of research, including the most pressing topics to be explored. Consequently, this paper aims to provide an agenda for future research on the combination of CR and in supply chains, derived from a systematic literature review.

As argued by Hoejmose et al. (2014), only a few studies consider the issue of CR across the supply chain context, with many only drawing on narrowly focused data or observations (Rajagopal et al., 2021). Wolf (2014) echoed this point and highlighted that more research should explore reputation and supply chains in combination to understand the linkages between them. At present, the academic literature provides interesting discussions, highlighting the overarching role of CR for business studies. However, there is a need to develop a theoretical foundation that will guide future research. Therefore, we undertake a literature review to address the following research goals:

1. Provide a state-of-the-art literature review, highlighting the historical development of the research field of CR.
2. Identify the most influential journals and authors which have shaped CR research.
3. Develop a current and consolidated definition of CR.
4. Highlight the importance of CR and its connection to the supply chain environment.
5. Outline an agenda for future CR research, relevant for supply chain topics.

For tackling the research goals, we divide this article into four parts. We begin with outlining CR and its connection to supply chain aspects, continue with the methodology section, before moving on to the evolution of CR as a research field and a conceptualization of CR. This provides the basis for a consolidated definition of CR. Then, we address the connection between CR and supply chain issues and highlight the importance of CR in a supply chain context. We conclude with a research agenda that can guide future CR researchers and practitioners to embark on their explorations in a targeted and structured approach.

### CR and Its Connection to Supply Chain Aspects

The market offerings, communications, and actions of a company’s supply chain partners pose a reputational risk, particularly for those operating in large supply networks as well as those involved in chains that span multiple countries where poor transparency, corruption, and human rights records are common. Thus, it is difficult to mitigate reputational risks in supply chains that are globally dispersed. Rajagopal et al. (2021) and Rajagopal et al. (2017) introduced the idea of looking at risk drivers from upstream and downstream supply chain partners, arguing that reputational risk is clearly overlooked in the supply chain literature. In addition, Dhingra and Krishnan (2021) explored social and environmental reputation costs along the supply chain and identified the importance of reputational risk sharing between supply chain partners. They highlight the lack of research in a supply chain context regarding reputation risk management and call for research to identify ways of substantially reducing reputational risks in supply chain settings. Mani and Gunasekaran (2018) echo these concerns, exploring how ethical behaviors and actions along global supply chains affect firm reputation. Their research highlights a need for further investigation of the role of reputation mechanisms in supply chain networks, influencing ethical and social actions, upwards and downwards the supply chain. Fan et al. (2021) document the risk of reputational spillover effects between supply chain partners. They recommend adopting a sustainability perspective when studying supply chains’ reputational risk. Likewise, Nguyen and Phan (2021) conclude that additional research is needed to explain reputational effects throughout supply chains and how to minimize reputational risks. Taking this further, Blom and Niemann (2022) argue that reputational risks along the supply chain have a predominant influence on a firm’s CR. However, despite the importance of this topic for practitioners and academics, the above authors found little literature on the topic. Reflecting on recent calls, further research is necessary to explore the topic of reputation in a supply chain context more holistically, including Corporate Social Responsibility (CSR) and environmental risks as influencing factors.

Dahlmann and Roehrich (2019) highlight that the engagement of an organization with its supply chain partners is crucial for the development of sustainable supply chains. This research field is complex because changes in a firm’s CR, resulting from the actions of one or more of its partners, can alter profoundly its relationships with other stakeholders. For example, during the Covid-19 pandemic, sales of internet-based fast fashion retailer Boohoo.com surged. However, in July 2020, newspaper reports identified that some of Boohoo’s suppliers paid employees below the minimum wage, and failed to follow appropriate social distancing guidelines (Thomond, 2020). An independent report, commissioned by Boohoo, found that the allegations were ‘substantially true’ (Levitt, 2020). In the wake of the controversy, several institutional investors sold their shares, denting Boohoo’s share price. PricewaterhouseCoopers (PwC) quit as its auditor and other leading accountancy firms ruled out working with the retailer.

A corporation’s reputation, as the Boohoo.com vignette illustrates, is co-created by organizations and their stakeholders. Therefore, CR is a dynamic construct, subject to external influences (e.g., customer perceptions) and is, thus, in a constant state of flux and development. Consequently, it varies in value over time (Veh et al., 2019). Hence, in this paper, we argue that CR matters in a supply chain context—a notion that has recently been heightened by developments in the EU. Specifically, EU businesses are facing increasing regulations concerning ethical sourcing and mandatory supply chain due diligence, forcing them to pay greater attention to the practices of their supply chain partners (European Parliament, 2021). Namely, the draft EU Directive on Mandatory Human Rights, Environmental and Good Governance Due Diligence envisages that companies falling within its scope will have to make appropriate efforts to identify their suppliers and subcontractors and implement actions to ensure that their business partners’ act in accordance with the company’s due diligence strategy. This includes measures relating to workload, occupational safety, working hours, exploitation, occupational health, fair trade, social compatibility, child labor, production of waste, and the sustainable use of natural resources (European Parliament, 2021). Other states and international organizations are also seeking to improve transparency in supply chains, especially in efforts to combat modern slavery (Australian Government, 2018; UK Parliament, 2015). The focus of CR is, thus, moving beyond the corporation’s own actions to also include those of their supply chain partners, posing the question as to how to manage CR within a supply chain context?

As the Boohoo.com case demonstrates, end-customers may not be the only actors shaping CR but it could be any stakeholder along the chain (Dewalska-Opitek & Bilińska-Reformat, 2021). Despite the current literature’s focus on the customer’s perspective, the scientific paradigm is highly likely to shift its focus toward a more comprehensive perspective (Bendixen & Abratt, 2007; Dahlmann & Roehrich, 2019; Jelinkova & Lostakova, 2016; Martin-de Castro, 2021; Panzone et al., 2016). This change is helpful when examining reputational spillover effects in a supply chain context. Following the argument of Petersen and Lemke (2015), one actor can utilize reputational triggers (i.e., offering, communication, and action) which may cause reputational aspects of the initiating actor to spill to others. For instance, ‘being innovative’ may spill from the supplier to the manufacturer when working with this supplier. These receiving organizations are *CR borrowers*, and the spill can happen willingly or unwillingly.

Between both owner and borrower are stakeholders that care about what is happening; they mediate the process. For instance, a supplier may employ children in the production process. As soon as the caring stakeholder is aware and perceives this action to be relevant (e.g., customers), it ‘reflects’ CR aspects directly from the owner to the borrower (e.g., from the supplier that employs children to the manufacturer that integrates this part in a wider system). This is a CR spillover, and the caring *CR reflector* is almost exclusively assumed to be the customer. However, the CR reflector could be any stakeholder who cares about what companies design and create, say, and how they behave (e.g., investors, policy makers, assessors, industry experts, communities, societies).

Recent global crisis heightened strains on supply chains, affecting CR. For instance, the Covid-19 pandemic placed enormous pressure on reputation management within global supply networks (Blom & Niemann, 2022). Many companies worldwide ran into difficulties, due to supply chain bottlenecks, as experienced, for example, at seaports, trade centers, and entire specialized economic zones (Phillips et al., 2022). Supply chains without any resource buffers, that were purposely designed for lean management, just-in-time, and cost optimization, showed little resilience during the Covid-19 pandemic (Phillips et al., 2022). Supply chain disruptions apparent during the pandemic were further exacerbated by the war in Ukraine. In the wake of such crises, many supply chains experienced domino and butterfly effects where small alterations caused large effects in complex systems (Hosseini & Ivanov, 2022; Yu et al., 2022). In response, many corporations sought to reintegrate sourcing and production into national and local regions.

In such an environment, reputation and supply chain management must be flexible and resilient enough to respond to global crises in real time. Consequently, corporations will need to continuously reassess their engagement with supply chain partners to assess and reduce risks. For mastering the risk challenge, an understanding of CR mechanisms is critical for supply chains and its corresponding stakeholders, as discussed earlier. However, literature on this topic is limited, in part because of CR and supply chains have been traditional regarded as separate ‘silos’ and due to data availability. Based on the high complexity of supply chain networks, companies do not always have a complete picture of their suppliers and sub-suppliers. Moreover, even if they possess the data, their willingness to share with the public (including research institutions) is limited, to preserve competitive advantage (Aamer et al., 2020; Quintana-García et al., 2021; Shaikh et al., 2020). Thus, empirical studies based on supply chain data are scarce. Nevertheless, understanding the mechanisms that co-create, transfer, and destroy CR along the supply chain is recognized as an important research topic (Marketing Science Institute, 2018; Syed Alwi et al., 2020). However, research on CR is fragmented across several disciplines and lacks a concerted supply chain perspective. To address this deficiency as well as to respond to recent calls for advancing CR research (Pérez-Cornejo et al., 2019; Veh et al., 2019), we conduct a Systematic Literature Review (SLR). When working with companies in CR, we recognize that the following areas are currently concerned with managing this topic: marketing, finance and accounting, general management, strategy, organizational studies, and supply chain management. The idea of this research paper originated from a business perspective, on the meso level (i.e., supply chain). To contribute to the currently underrepresented literature, due to data availability, practitioners’ insights offer new perspectives and knowledge in the field (Aguinis et al., 2022; Schön, 2017; Stokes, 2011). During the development of this research project, and acknowledging its relevance for supply chain topics, we realized that CR is not very well featured in the supply chain literature domain. Therefore, before discussing CR in relation to supply chain aspects, it is important to have a clear view of the CR literature. In this article, we continue with the methodology of our SLR and bibliometric mapping. This leads us to a consolidation of existing CR definitions.

### Systematic Literature Review (SLR)

To provide an overview of the literature and develop a consolidated definition of CR, we carried out a SLR of CR research. A SLR is a powerful means for detecting and making sense of conceptual as well as methodological issues (Boell & Cecez-Kecmanovic, 2015; Crossan & Apaydin, 2010; Grewal et al., 2018; Sarmento & Simões, 2018; Veh et al., 2019). It is also suitable for identifying critical areas for further research and informing theory development (Köhler et al., 2017; Kohtamäki et al., 2018). Hulland et al. (2018) highlight the demand for empirical studies to systematically detect and better understand specific research areas and their gaps, as well as their future research potential. The aggregation of studies from different disciplines allows us to attain a comprehensive overview of the body of knowledge, and to highlight the inter-relationships of various constructs, research areas, and broader literature fields (Bier et al., 2020; Burgers et al., 2019).

**Fig. 1:**
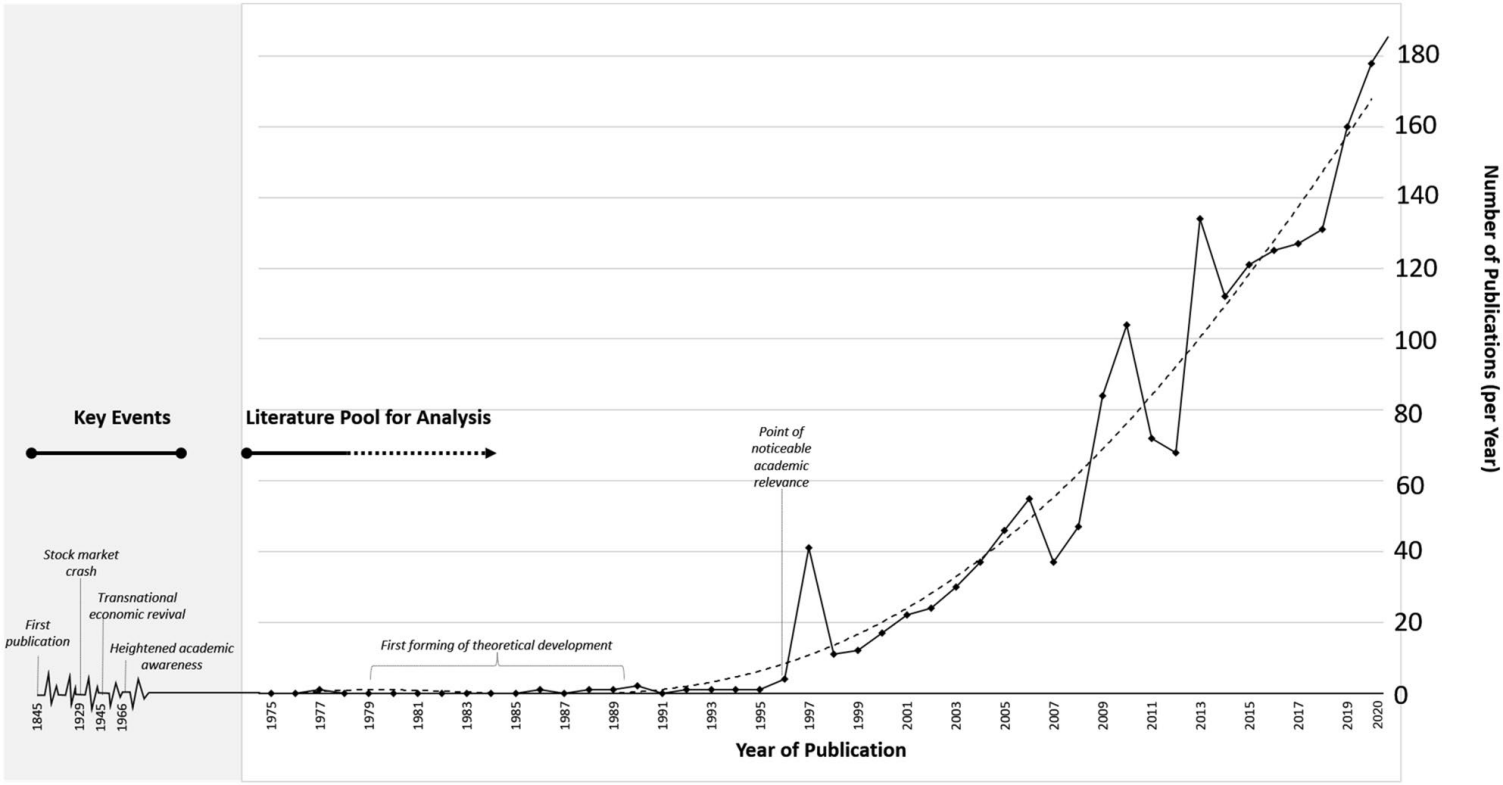
Key events of corporate reputation and related publications per year (1975–2021)

The literature review process began with a planning phase, including the development of inclusion/exclusion criteria for the selection of published materials (Grewal et al., 2018; Kohtamäki et al., 2018). The study focuses solely on scientific peer-reviewed articles in top tier journals. We only reviewed English contributions, published in journals listed in the ABS Ranking (that also includes FT50 journals). Specifically, we took the ABS ranking as a guidance framework and limited inclusion to papers published in journals ranked ABS2 to ABS4*. Our intention is not to downplay non-English or low/unranked articles. We rather sought to identify a literature pool that has greater potential to be highly cited. Setting a recent timeframe is recommended by Hox et al. (2017), to reveal the current state of the art and research directions within a field. Our data set includes articles published between 1996 and 2021. Prior to 1996, the CR literature was limited and the number of publications on CR substantially increased from less than 2 to over 40 per year (see Fig. 1).

### Coding

The SLR followed the procedures recommended by Sarmento and Simões (2018). In the first stage, robust citation index services were identified. Scopus, produced by Elsevier, allows a subject search with citation tracking in the sciences and social sciences with over 69 million records (Scopus, accessed on 17.04.2021). This, in combination with Web of Science, generated more than 90 million records (Web of Science, accessed on 17.04.2021). The overall number of publications on Google Scholar containing the exact phrase ‘corporate reputation’ in the title, abstract, or keywords is approximately 72,500 (date: 17.04.2021). Besides Google Scholar, the search was also conducted in the Web of Science, EBSCO, Social Science Research Network (SSRN), and Scopus databases for identifying articles dedicated to ‘corporate reputation.’ There is a noticeable publication uptake in 2011 (Fig. 1), which served as a suitable starting point for further assessment. Working with the literature of the past ten years ensured that we capture the current understanding of CR. This initial sample contained 1922 articles, suitable for our bibliometric mapping analysis. For the SLR, the titles, abstracts, and key words of the 1922 relevant articles were examined for relevance to the topic ‘corporate reputation.’ This was the second stage. In some cases, although the title and key words appeared promising, the content of the abstract was of little relevance for our SLR. These articles were discounted, following Kohtamäki et al. (2018), who argued that generic articles with no particular contribution to the research question should be excluded. This filtering reduced the dataset from 1922 peer-reviewed articles to 235 scientific papers relevant for the study.

In the third stage, coders searched for the phrase ‘corporate reputation’ in every article, using the electronic search function. Two coders worked through the 235 articles independently. The content of the 235 articles relating to CR was selected by the coders and then transferred to an Excel spreadsheet. Each coder indicated whether they regarded the content as relevant or not. Discrepancies between markers, based on their individual assessments, were discussed and agreement reached. Two weeks later, this procedure was repeated. Only those articles regarded as relevant on both occasions by both coders proceeded to the next coding phase. This process ensured consistency in the classification of articles, suitable for further analysis.

In the third stage of coding, the content of the articles where CR was explained, defined, or distinguished from other concepts was highlighted. Coded sentences or paragraphs were transferred into a new Excel sheet. The extracted sections and definitions identified by both coders were then compared. For 37 text phrases where the two coders disagreed, they reached agreement through a negotiation process. In eight cases, the two could not come to an agreement, so these text passages were discarded and not considered further in the process. Overall, the two coders identified and agreed on over 583 text passages suitable for the next stage of coding.

In the fourth stage of coding, within the selected text blocks, words and short text phrases that dealt directly with CR were identified. We adopted ‘descriptive coding’ to develop “an inventory of topics for indexing and categorizing” (Miles et al., 2019, p. 65). In the initial coding of the text block data, highlighted text chunks represented distinct meanings, which is typical in ‘first cycle coding’ (Saldaña, 2016). The text passages were printed twice on separate cards. Each coder worked separately with the identical card set, allocating the highlighted text chunks to meaningful categories. In the ‘second cycle coding’ step, the coding material was then categorized, following the principles of ‘pattern coding’ (Saldaña, 2016). Cards containing more than one code were categorized in multiple ways, a process known as ‘simultaneous coding’ or ‘double coding’ (Saldaña, 2016). The two coders’ classification of manually categorized cards (i.e., highlighted text chunks) were copied into an Excel spreadsheet, and an inter-coder reliability index computed. The two coders discussed any disagreements, as part of the negotiation process.

In qualitative research that explores rich interview data, inter-coder reliability tests could be repeated multiple times, resulting in an eventually high level of agreement between coders (e.g., Campbell et al., 2013; Lemke et al., 2011; MacPhail et al., 2016). In our study, the inter-coder agreement was 94.3% after just one coding round. Agreement by chance is eliminated by a Cohen’s Kappa of 91.3%, which exceeds substantially the recommended threshold (88.4%), as suggested in the literature (Cohen, 1968; Lombard et al., 2002; Perreault Jr & Leigh, 1989). Both results may not be surprising, given that the coded text were existing definitions and CR descriptions in academic publications, which were intended to be clear and precise, leaving little room for ambiguity and subjective interpretation. The resulting words and text passages and content gathered in this analytical stage provided the basis for formulating a holistic definition of CR. Although CR has been a research topic for over four decades, the understanding of the term has evolved in different sub-disciplines, resulting in fragmented perspectives (Gomez-Trujillo et al., 2020; Khan & Digout, 2018).

### Corporate Reputation as a Research Field

The origins of research on CR are mostly USA-based, with the stock market crash of 1929 laying the foundations for an awareness of CR on a broader scale (Jones et al., 2000; O'Neill, 1977; Stevens, 1975). During the following decade, due to several corporate scandals based on discrimination against women, Jews, African Americans, and other minorities, the US government began to curtail unethical behavior and to restrain the power of corporations (O'Neill, 1984). Consequently, in the 1930s, a new system of regulations and regulating institutions emerged in the US. Following US military occupation after World War II, several regulatory standards were transposed and influenced standards for transnational companies across Western Europe (Maier, 1977; Majone, 2002). From the mid-1960s onwards, a slowly increasing number of publications on the topic indicate a rising awareness in academia—CR turned into a public issue. Figure 1 shows the distribution of peer-reviewed publications on the topic of CR, from 1975 until 2021. Given that the number of publications continues to rise, it seems unlikely that the research field has yet reached a peak, especially given the growing public awareness about CR and its media coverage (Fragouli, 2020; Gatzert, 2015; Money et al., 2017; Veh et al., 2019).

The mid-1970s witnessed a heightened interest in CR among academics, as the post-war consensus on business-state relationships in western societies dissolved. Specifically, Friedman (1970) and other Chicago School economists prompted debate on whether businesses were over-regulated to the detriment of macroeconomic performance. They argued that a company’s only responsibility was to its shareholders, while adhering to the legal system in which they operated. In the 1980s, the development of CR as a scientific topic began, utilizing theoretical approaches from business economics. In this context, CR theory was founded on game, signaling, and stakeholder theories (Weigelt & Camerer, 1988). In the 1990s, sociological perspectives informed academic perspectives on CR, drawing on organizational and social identity theories (Walker, 2010).

**Fig. 2:**
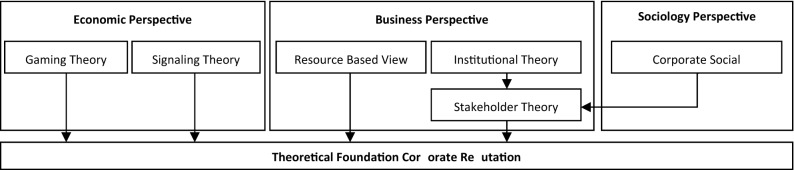
Theoretical Foundation of Corporate Reputation Research in the 1980s and 1990s

The origins of CR as a research subject are multi-theoretical. Historically, many prominent theoretical contributions come from game and signaling theory (Fombrun & Shanley, 1990; Rindova et al., 2005; Veh et al., 2018). This emphasizes that CR serves as a signal of a firm’s credibility attributes, products, or services (Saxton, 1998; Shapiro, 1983). In addition, Weigelt and Camerer (1988) connect game and signaling theory and outline how reputation can emerge from the past actions and behaviors of a firm. Turban and Greening (1997) combine the concepts of social identity and signaling theories to develop the concept of social performance as an aspect of CR. Johnson and Greening (1999) elaborate on this with the idea that good social performance enhances a firm’s overall reputation. Thus, proactive CSR creates a reputation that a firm is reliable and honest, and signals to customers (Sethi et al., 2016) that the corporation offers a superior product and service quality (Mishra et al., 1998; Purohit & Srivastava, 2001; Rao et al., 1999). Fombrun (2005) argues that the problem of a concrete definition regarding the concept of CR stems from diverse studies, which examine the construct of CR from different disciplinary perspectives. Both highlight the need for an integrated view. Thus, the Integrative School of Thought was born. Figure 2 provides a snapshot of the theoretical foundation.

Barney (1986) and Dierickx and Cool (1989) developed CR theory from a Resource-Based-View (RBV) perspective. Accordingly, CR is used for developing an advantage over competitors, and Hall (1992) emphasizes that CR can differentiate a company from its competitors. Shielding reputational barriers can hinder competitors’ entry to a market or an industry where an existing company’s reputation is strong. Overall, organizational strategists consider CR a competitive and, thus, strategic asset to distinguish a company from its competitors (Rindova & Fombrun, 1999).

DiMaggio and Powell (1983) and Meyer and Rowan (1977) use institutional theory to inform their stream of CR research. This influenced the work of Staw and Epstein (2000) on how CR emerges in organizational interactions. Rindova et al. (2005) redefined the idea of CR as a social construct derived from the collective awareness and acceptance of an organization in its stakeholder environment. Referring to the theoretical concept of CR, CSR, and stakeholder theory, Mitchell et al. (1997) mapped out the connection between CR and CSR. Considering these findings, a conceptual basis for empirical studies was formed. The aim was to demonstrate how corporate social performance is linked to different corporate performance indicators, i.e., CR (Brammer & Pavelin, 2006; Turban & Greening, 1997).

Fombrun and Van Riel (1997) draw on sociological perspectives and stakeholder theory. They explored the connection between social constructs, such as rankings and reviews, and considered their influence on relationships between organizations and their stakeholders. Granovetter (1985) and White (1981) point out how social rankings and reviews strongly influence stakeholders’ perceptions of CR. Thus, CR represents an aggregated assessment of a firm from the perspective of both its stakeholders and their peer groups. Consequently, although CR is difficult to imitate for other companies (Fombrun & Zajac, 1987), it essentially is a perception that is largely outside the direct control of the organization.

#### Analogous School of Thought

The theory of reputation and its definition derives from psychology. The concept of self-identity thus informed the creation of reputation as a research field. Martineau (1958) defines corporate image as a sum of functional qualities and psychological attributes that exist in the mind of the consumer. This view is mainly influenced by the idea of reputation as a behavioral construct as part of self-identity theory. However, Kennedy (1990) argues that corporate image is synonymous with CR. Early studies stemming from the Analogous School of Thought focused on the concept of corporate image rather than on CR. The choice of terminology was a child of its time. In the 1960s and 1970s, corporate image research was very fashionable, while the term CR had not yet been established. Rindova (1997) notes that those authors from the Analogous School of Thought largely have a background in public relations and have, therefore, focused on the concept of corporate image rather than CR. As a result of the research undertaken by this school of thought, many regard the terms corporate image and CR as identical. Hence, ambiguity about the conceptualization of CR persists.

#### Differentiated School of Thought

Authors from the Differentiated School of Thought consider CR and corporate image as two different but interrelated theoretical concepts. This approach generated two ideas. Firstly, a firm’s reputation is one layer of a corporate image. While, secondly, CR is influenced by multiple images perceived by a company’s stakeholders. Many authors of this school (Bromley, 2001; Fombrun, 1996; Fombrun & Shanley, 1990; Gray & Balmer, 1998; Rindova, 1997; Saxton, 1998) argue that CR reflects a firm’s image over time perceived by its stakeholders. It is shaped by the thoughts and words of its stakeholders. In addition, Fombrun (1996) suggests that CR is essentially backwards looking, characterized by customers experiences created in the past.

#### Integrative School of Thought

**Fig. 3:**
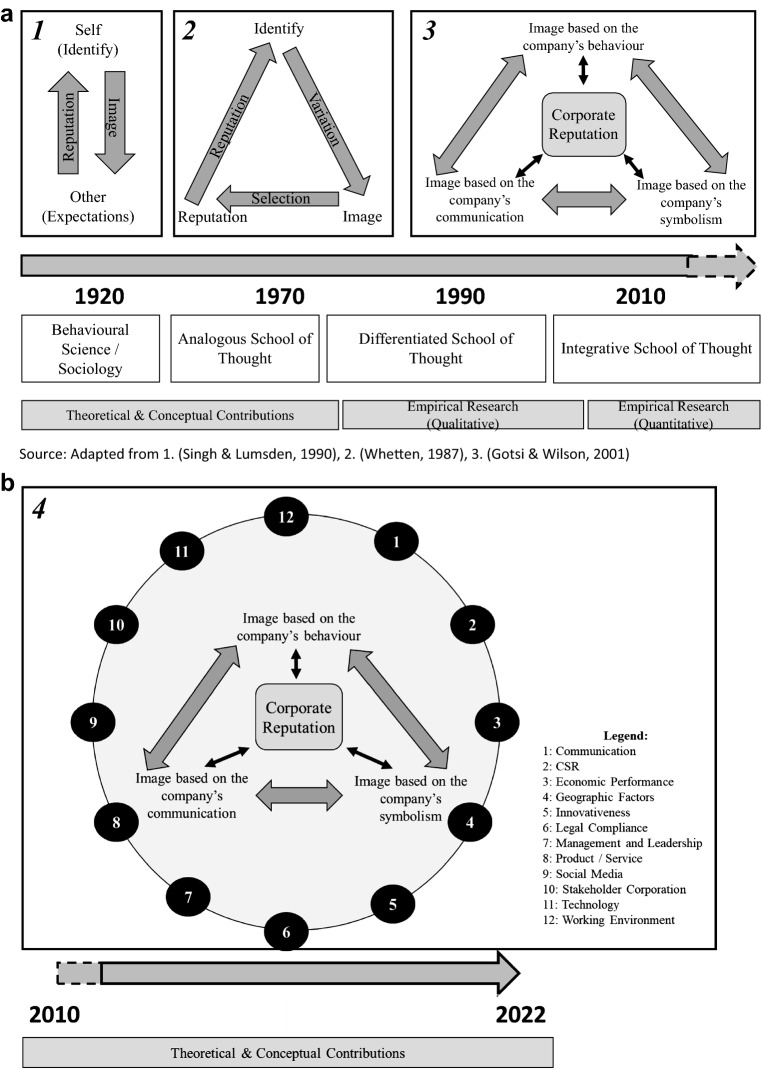
**a** School of Thoughts in corporate reputation research history. *Source*: Adapted from 1. (Singh & Lumsden, 1,990), 2. (Whetten, 1,987), 3. (Gotsi & Wilson, 2,001). **b**: Consolidated School of Thought

Authors from this school argue that a bilateral dynamic relationship between a firm's reputation and its projected corporate images build the foundation of a company’s reputation. Thus, CR is not static and needs to be constantly managed with planned, formal, sensitive, and target-oriented communication activities. They define CR as an umbrella construct which includes different layers: corporate image, organizational identity, organizational culture, and stakeholder perceptions of past behavior and action (Cian & Cervai, 2014). Thereby, CR is rooted in both internal and external stakeholder groups which are influenced in their perception of CR by the company’s image, identity, culture, and communication activities. However, the conceptualization of CR remains debatable, and Walker (2010) argues that researchers across disciplines need to be open to new concepts and definitions. The historical development of CR is outlined in Fig. 3a.

#### Consolidated School of Thought

The integrative school of thought regards CR as the expectation of stakeholders toward the company’s future actions to secure CSR aspects as well as to show true engagement in sustainability along their corporate value chain system. In this sense, CR is not merely backwards oriented—it is rather the trust that stakeholders place in companies when it comes to fulfilling their promises and adhering to the values they communicate. This includes the traceability and transparency of their value chains. Since the late 2000s, climate, environmental, and sustainability factors have increased the pressure for companies to focus more on conservation aspects of their CR. Additionally, Dahlmann and Roehrich (2019) point out that the engagement of an organization with its partners along its supply chains is crucial for the development of long-term sustainability and to ensure green and sustainable supply chains in the future.

The prevailing view in the contemporary CR literature derives from a focus on end-consumers (Dijkmans et al., 2015; Kiessling et al., 2016; Quintana-García et al., 2021; Walsh et al., 2014; Wies et al., 2015), which continues to endure (Brønn & Brønn, 2017; Camilleri, 2017; Walsh et al., 2018). It is surprising to see that the end-consumer perspective still serves as a reference point for directing and guiding the reputational debate, given that CR is created, shaped, interpreted, and is meaningful throughout the entire chain of business’s operations (Guo et al., 2020; Quintana-García et al., 2021).

### Consolidated Definition of CR

Definitions providing new insights and contemporary knowledge were coded manually before we entered them into Excel. At first, we searched the entire paper, using our search term, ‘corporate reputation.’ In doing so, we identified the relevant paragraphs in which definitions appeared and read these carefully. In the analysis, we did not work with a pre-defined list of codes that has the risk of losing important information. Rather, two independent researchers coded the definitions stemming from the research papers and compared them. They then ranked the grouped content of the CR definitions according to frequency, identifying the most important and most frequently mentioned terms and incorporating them into the definition. The goal was not to go into as much detail as possible and to characterize the individual underlying foundations of CR, but to look for definitions that had a high degree of similarity with each other and could, thus, be consolidated. Our synthesized definition of CR derives from the 235 coded articles, of which 183 or 77.87% have been incorporated or reflected in the consolidated CR definition proposed in this paper. We, thus, have a solid basis that offers a contemporary definition of CR to provide researchers and practitioners with common ground for conceptualizing CR.

Corporate reputation is a unique, intangible, status-based asset, emerging from the stakeholders’ perception of the firm’s future commitments and how closely it previously acted within the overall expectations of its stakeholders, based on their beliefs and values. This is judged by their evaluation of future commitments and past experience with the company (i.e., prior actions, performance, and behavior). The perception represents the aggregated opinion of the stakeholder community and is co-created by the interplay of organizations, their stakeholders, and the competitive environment.

### Bibliometric Mapping—Identifying Key Clusters in the Current CR Literature

In order to identify the most important journals and influential CR authors, as well as to identify the key dimensions of CR, we undertook a bibliometric analysis (Singh & Dhir, 2019). This analysis includes a variety of techniques that are used to support a SLR (Fellnhofer, 2019; Gurzki & Woisetschlaeger, 2017; Samiee & Chabowski, 2012; Vogel, 2012). Bibliometric visualization is a comprehensive method to identify the most influential authors in a research domain along with the most important topics associated with it (Fellnhofer, 2019; Ji et al., 2020; Leydesdorff et al., 2016; van Eck & Waltman, 2017). Small (1973) introduced co-citation analysis as an effective tool to highlight the interlinkages between different knowledge fields and their underlying intellectual structure. The VOSviewer mapping technique works with co-citation linkages between authors and key words (Meng et al., 2020; Van Eck et al., 2010). This allows for plotting networks and citation maps to visualize the relationships between diverse topics, publications, authors, or other items of interest.

**Fig. 4:**
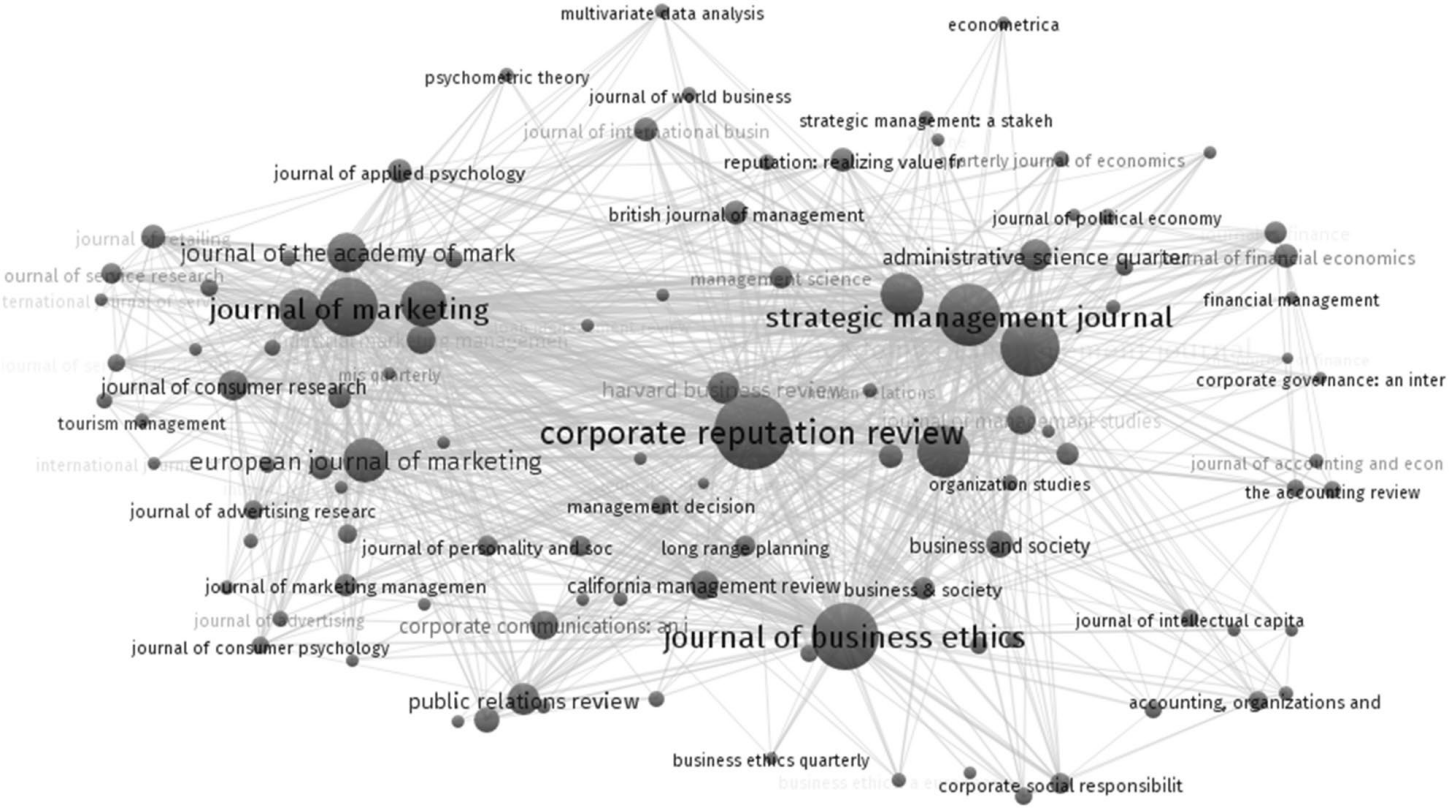
Bibliometric mapping of journals for the topic corporate reputation (1975–2021)

For defining CR, we worked with our smaller set of 235 articles (Table 1). For creating a bibliometric map, however, we wanted to display a more holistic view that displays the connections between networks of CR studies. For the latter, the dataset of 1922 suitable articles was merged into a comma-separated value file (CSV) and imported as tabulated data into Microsoft Excel. In a second step, network maps were generated to visualize the co-citation analysis and highlight the most influential authors in the CR field. We used the visualization program VOSviewer, version 1.6.14 (VOSviewer, accessed on 17.04.2021), to perform this analysis. The input file was used by the VOSviewer algorithm to locate items in a low-dimensional space. This was necessary to define the distance between sets of items as an indicator of their relatedness. Publications are concentrated in the following journals: Corporate Reputation Review, Journal of Marketing, Journal of Business Ethics and Strategic Management Journal (Fig. 4). Based on the total number of 1922 publications in high impact journals, CR developed from a niche topic into one that is of general interest. The specialized Corporate Reputation Review is the dominant publication outlet and is placed at the center of the bibliometric map. Its aim is to be the main communication platform for CR research (Fombrun & Van Riel, 1997).

**Table 1:** Data set overview

Publication date	Number of articles	% of Total articles
Before 2008	49	20.85
2008	2	0.85
2009	10	4.26
2010	13	5.53
2011	9	3.83
2012	11	4.68
2013	14	5.96
2014	12	5.11
2015	13	5.53
2016	17	7.23
2017	19	8.09
2018	12	5.11
2019	19	8.09
2020	21	8.94
2021	14	5.96
Total:	235	100

Research Area	Number of Articles	% of Total articles
General Management	83	35.32
Marketing	74	31.49
Strategy	25	10.64
Finance and Accounting	22	9.36
Operations & Supply Chain Management	17	7.23
Organizational Studies	14	5.96
Total:	235	100

Method	Number of articles	Distribution
Case Study	23	10.21
Conceptual	37	18.30
Qualitative	19	9.36
Quantitative	103	45.11
Mixed Methods	39	17.02
Total:	235	100

**Fig. 5:**
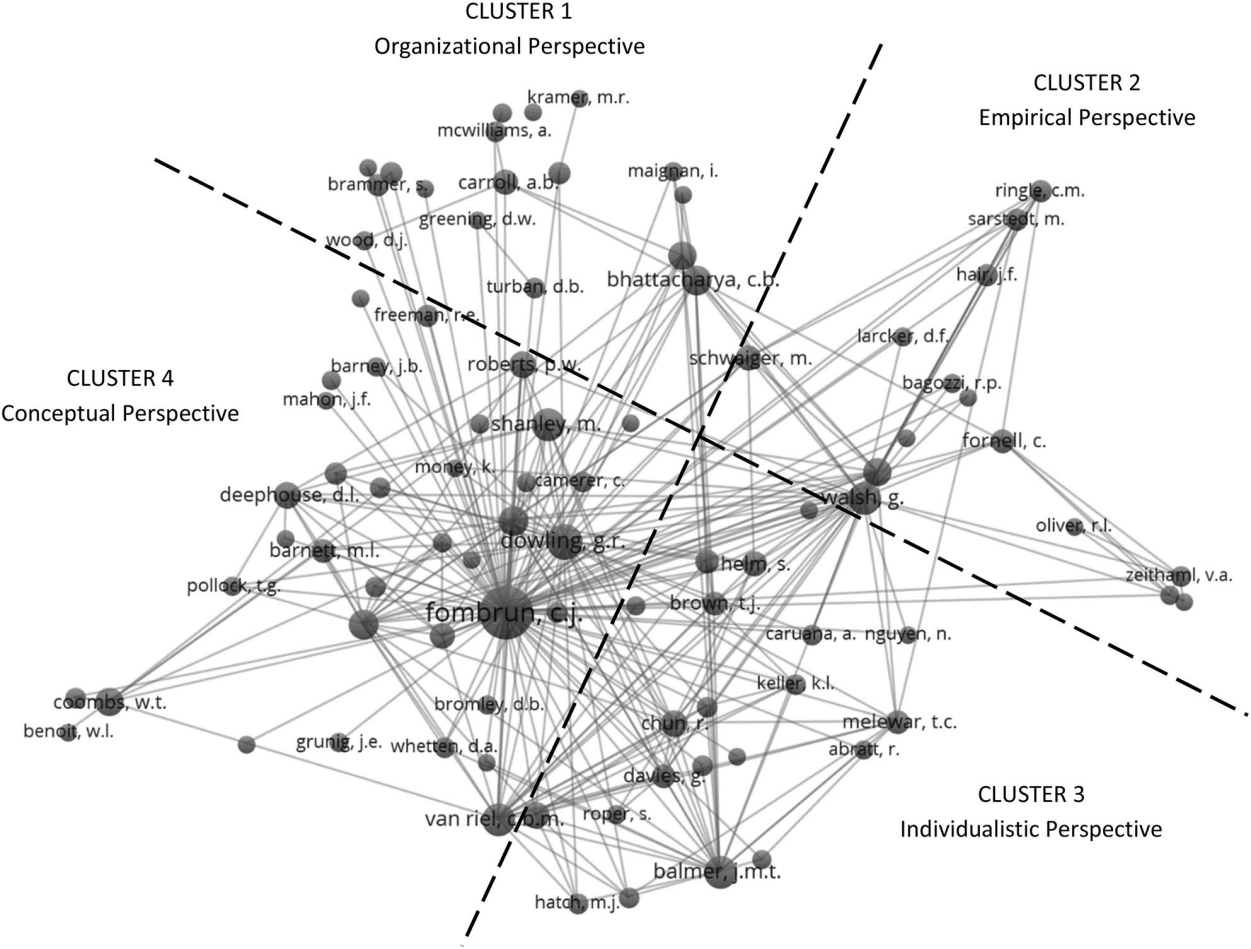
Co-citation map of the research field corporate reputation between 1975 and 2021

In the network analysis, a dot represents a journal and dot sizes indicate the volume of publications on the CR topic. The analysis also illustrates the proximity of journals, based on co-referencing frequency. When working with the 1922 publications, we identified the most cited CR authors between 1975 and 2021, grouping authors with fifty or more citations, and plotted co-citation maps (Fig. 5).

The dotted lines demarcate four clusters, each with a center point indicating the leading author. This author has been cited most often by related authors in the cluster space, signaling the lead author’s influence—or contribution—to the work of others. The clusters are clearly distinguishable while still being visually interconnected. The four clusters capture influential researchers in the field of CR and different research directions. The typology can provide researchers with an orientation to the CR topic. Consequently, it can thus help researchers plan their own future investigations.

#### Cluster 1—Organizational Perspective

Researchers from this cluster connect CR with relationship marketing and CSR (Hildebrand et al., 2011). Most publications appeared between the years 2000 and 2015 and their geographical setting is principally Germany, USA, and Australia. Most authors in this cluster come from the fields of marketing and organizational studies. Against this background, the research cluster typically has the customer-company relationship as a dyadic focus and explores how sustainability, social, and ethical aspects influence CR in a diverse stakeholder environment (Bhattacharya et al., 2010; Brammer & Pavelin, 2005, 2006; Greening, 1995). The research design of papers often follows those employed in organizational theory and organizational psychology (Cable & Turban, 2003; Einwiller et al., 2010). Consequently, multiple researchers specializing in human resources contribute to this cluster and studies are usually conducted from the perspective of employees (Cable & Turban, 2003; Greening & Turban, 2000). Surveys, event studies, and experiments are preferred for empirical analysis.

#### Cluster 2—Empirical Perspective

CR research in Cluster 2 consists of papers mostly by German marketing academics. Research forming this cluster is typically data driven and part of performance marketing (Raithel & Schwaiger, 2015). Studies often draw on German or European samples of respondents and companies (Schwaiger et al., 2010). Since 2000, the researchers have used Structure Equation Modeling in customer-based reputation research (Schloderer et al., 2014) to understand how CR is associated with customer satisfaction, loyalty, and trust (Walsh et al., 2014). Researchers from this cluster are also interested in the development and utilization of other regression-based statistical methods in marketing research (Schwaiger, 2004; Wilczynski et al., 2009).

#### Cluster 3—Individualistic Perspective

CR researchers in Cluster 3 were most active in the years 2000 to 2010. The majority are UK-based academics, and their research relates to the fields of marketing and consumer behavior. They sought to explain CR with findings from organizational research, drawing on concepts from social identity and corporate branding theories (Balmer, 2008; Balmer & Greyser, 2006; Melewar, 2003). These scholars wrote seminal papers, separating the concepts of corporate identity, corporate image, corporate branding, and CR (Abratt & Kleyn, 2012). Increasingly, topics from the field of social media marketing and digital marketing attracted attention, such as a consideration of e-reputation (Chun & Davies, 2001). Overall, this cluster focuses on CR as a customer-centric concept (Walsh et al., 2015).

#### Cluster 4—Conceptual Perspective

Cluster 4 is almost exclusively dominated by US-based scholars, active since the 1990s (Abratt & Kleyn, 2012; Barney, 1991; Deephouse & Carter, 2005; Fombrun & Shanley, 1990; Fombrun & Van Riel, 1997). Articles explore the topic of CR often on a sectoral basis, beginning with the fashion industry and the banking sector (Fombrun, 1995; Preece et al., 1995). Based on the findings generated from these industries, the first empirical studies attempting to estimate the effects of CR on financial performance emerged in the early 2000s (Barnett et al., 2006; Roberts & Dowling, 2002). Later, additional dimensions were added such as product and service quality, leadership performance, and CSR (Barnett, 2007). The authors in this cluster laid the foundations for CR as a distinctive field of research. They developed measures of CR which have since been adapted and further refined (Fombrun et al., 2000, 2015; Ponzi et al., 2011). In terms of theory, most of the initial published research is based on signaling and stakeholder theories (Baumgartner et al., 2020), as well as those related to crisis and communication management (Coombs, 2020). The table below contrasts and compares the theories applied in the four clusters, listed in order of popularity:

As Table 2 shows, Cluster 1 has a greater CSR focus which we also find in the theories applied. Cluster 2 is almost exclusively concerned with the end-consumer which explains the preference from working with theories stemming predominantly from marketing. Cluster 3 adopts an individualistic perspective and works with the theories that shed light on individual actors and their identities. Cluster 4 typically explores questions around theory development and methods of measurement (Table 2).

**Table 2:** Theories and Topics dominant in the relating research clusters

Organizational perspective (Cluster 1)	Empirical perspective (Cluster 2)	Individualistic perspective (Cluster 3)	Conceptual perspective (Cluster 4)
CSR and relationship marketing CSR Theory consumers-corporations’ relationships Customer satisfaction and CSR The role of CSR in corporate communication CSR and reputation, CSR as a corporate competitive advantage Organizational identification / CSR and Identity theory Marketing and CSR CSR and customer relationship building CSR and stakeholder theory Sustainability CSR as an organizational strategy Market based view Organizations and CSR communication CSR and Employee branding Corporate marketing perspective and reputation	Service marketing and customer-based view customer view perspective client-based view, stakeholder theory, stakeholder approach, integrative school of thought customer-based reputation, relationship marketing information processing and social role theory, Consumer behavior theory dyadic model building customer firm relationship transfer of theories into the web-based space research in online marketing Online retailing and user experience etc customer online experience from 2000 also social media word of mouth theory customer communication	Corporate Identity and Corporate Reputation Identity theory Social identity Identity based view, social identity, and image theory Brand and identity view Ethical marketing / ethical corporate marketing Stakeholder theory Value based identity Employee branding / reputation ethical corporate identity consumer behavior the individual and the organization customers and employees employee loyalty employee view on corporate reputation	Reputation and strategic perspectives Reputation measurement Reputation reviews and analysis Social construct theory Conceptualization of measuring corporate reputation Conceptual model building Definitions of CR Strategic perspective on CR Overviews and Analysis of CR Theory building

## The Importance of CR in the Supply Chain

As witnessed in the Boohoo.com case, the CR of firms in a supply chain is interconnected. Often, suppliers must adjust their own strategies to fit with the business concept (and thus, intended CR) of manufacturers or retailers (Hoejmose et al., 2014; Petersen & Lemke, 2015; Quintana-García et al., 2021). Thereby, CR frames the process of how stakeholders obtain superior value from their supply chain partners.

**Fig. 6:**
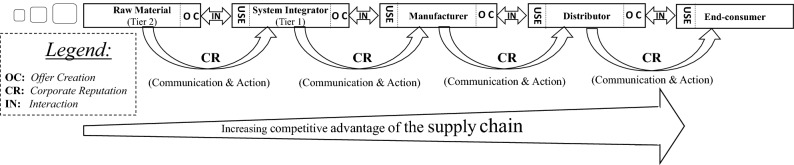
Corporate reputation in a simplified supply chain context. *Source*: Based on Lemke and Petersen (2013)

Within the supply chain context, CR has the potential to strengthen stakeholder attachment and commitment to a corporation. For example, suppliers adjust their behavior and management ethics toward their downstream customers to ensure that they are in the position to make the value proposition for their buyers stronger. Consequently, CR parallels the flow of micro-interactions and exchanges of offerings serve like a tier-to-tier baton that contributes to the competitive advantage of an entire supply chain. When the offering is ‘in use’ (e.g., a tier 1 supplier obtains raw material), a new offering becomes created (e.g., for the manufacturer). At the risk of simplification, Fig. 6 introduces the concept in a generalized supply chain setting.

Figure 6 presents a linear input-through-output process of a supply chain, where CR is formed along the supply chain. Resource integration happens at each stage of the chain and the smaller squares on the left symbolize the beginning at the raw material stage. Consequently, CR becomes part of designing a new offering for the next chain member, which becomes larger, more substantial, complete, and tailored toward the needs of the end-consumer market. We indicate this in the form of the increasing ‘competitive advantage’ that all supply chain members co-create.

The analysis of the literature resulted in the identification of twelve dimensions of CR, and Table 3 provides an overview in alphabetical order:

**Table 3:** Corporate reputation: dimensions

Dimension	Description	Example sources
Communication	Media coverage / use of all media channels available Use of acoustic and visual footage Transparent Up-to-date reporting Presentation of facts and figures	Barnett, M.L., & Leih,S. (Kohtamäki et al.), Coombs, W.T. (2007), Einwiller, S. A., Carroll, C. E., & Korn, K. (2010), Ji, Y. G., Tao, W., & Rim, H. (2020), Köhler, C., Mantrala, M. K., Albers, S., & Kanuri, V. K. (2017)
CSR	Ethical / social responsibility Environmental conservation Fair and sustainable production / fair trade	Barnett, M.L. (2007), Bhattacharya, C., Smith, N., & Palazzo (2010), Brammer, S. J., & Pavelin, S. (2006), Greening, D. W., & Turban, D. B. (2000), Hildebrand, D., Sen, S., & Bhattacharya, C. (2011), Kiessling, T., Isaksson, L., & Yasar, B. (2016), Vanhamme, J., & Grobben, B. (2009)
Economic Performance	Financial performance / stability / profitability Competitive market performance compared to its competitors Growth potential Financial risk management	Barnett, M.L. (2007), Fasaei, H., Tempelaar, M. P., & Jansen, J. J. (2018), Gatzert, N. (2015), Jones, G. H., Jones, B. H., & Little, P. (2000), Love, E. G., & Kraatz, M. S. (2017), Roberts, P.W., & Dowling, G. R. (2002), Thakor, A. V. (2015)
Geographical Factors	Sustainable sourcing Country of Origin effects Cultural and geographical issues	Hoffmann, N. C., Yin, J., & Hoffmann, S. (2020), Ingenhoff, D., Buhmann, A., White, C., Zhang, T., & Kiousis, S. (2018), Tannous, K., & Yoon, S. (2018)
Innovativeness	First to market Ability to create trends Ability to adapt to change Ability to find new sustainable raw materials or substitutes	Frombrun, C. J., Ponzi, L. J., & Newburry, W. (2015), Sarmento, M., & Simoes, C. (2018), Sridhar, M., & Mehta, A. (2018), Karamchandani, A., Srivastava, S. K., Kumar, S., & Srivastava, A. (2021)
Legal Compliance	Legal risk management Legal standards and predictability of legal decisions Bribery and fraud avoidance Compliance with the law and contracts	Lemke, F., & Petersen, H. L. (2013), Lemke, F., & Petersen, H. L. (2018), Azadegan, A., Syed, T. A., Blome, C., & Tajeddini, K. (2020), Baah, C., Jin, Z., & Tang, L. (2020), Baumgartner, K. T., Ernst, C. A., & Fischer, T. M. (2020), Busse, C., Meinlschmidt, J., & Foerstl, K. (2017)
Management and Leadership	Trust / Credibility / Responsibility of the Leaders / Managers Clear and transparent management vision and agenda Risk awareness / risk prevention	Busse, C., Meinlschmidt, J., & Foerstl, K. (2017), Fragouli, E. (2020), Dhingra, V., & Krishnan, H. (2020)
Product / Service	Quality / Value for money Customer needs / needs fulfillment	Shapiro, C. (1983), Walsh, G., Bartikowski, B., & Beatty, S. E. (2014), Walsh, G., Beatty, S. E., & Holloway, B. B. (2015), Walsh, G., Schaarschmidt, M., & Ivens, S. (2018)
Social Media	Social media performance Social Media visibility / coverage Social media content & communication management Social media engagement with stakeholders	Colicev, A., Kumar, A., & O`Conner, P. (2018), Dijkmans, C., Kerkhof, P., & Beukeboom, C. J. (2015), Ott, L., & Theunissen, P. (2015), Ajayi, O. A., & Mmutle, T. (2021), Coombs, W. T. (2020)
Stakeholder-Corporation Interaction	Stakeholder community integration in decision making processes Networking with the stakeholders Feedback speed / feedback frequency	Barnett, M.L. (2007), Quintana-Garciá, C., Benavides-Chicón, C. G., & Marchante-Lara, M. (2020), Wies, S., Hoffmann, A. O., Aspara, J., & Pennings, J. M. (2015)
Technology	Data security / data protection Access to knowledge and company information IT System and access stability	Petersen, H. L. & Lemke, F. (2015), Ponzi, L. J., Fombrun, C. J., & Gardberg, N. A. (2011), Karamchandani, A., Srivastava, S. K., Kumar, S., & Srivastava, A. (2021), Mani, V., & Gunasekaran, A. (2021)
Working Environment	Human rights / child labor Diversification and equal pay Staff fluctuation / staff satisfaction Occupational safety / well-being	Chun, R., & Davies, G. (2010), Greening, D. W., & Turban, D. B. (2000), Turban, D. B., & Greening, D. W. (1997), Schaarschmidt, M., & Walsh, G. (2020), Sims, R. (2009), Singh, K., & Misra, M. (2021)

The dimensions of CR displayed in Table 3 represent an additional pillar of CR that must be included in a holistic discussion of CR today. Regarding the dimensions, we further developed an idea of a Consolidated School of Thought from our historical analysis of CR (see Fig. 3b). This school views CR on a broader canvas—one that is embedded in the stakeholder environment, framed by the dimensions derived from our research. We believe that this understanding must be included in a modern and consolidated version of CR. Thus, a theoretical model should reflect the dimensions of CR to meet the requirements of contemporary and preventive reputation management in the stakeholder environment of any business organization.

We aim to identify the emerging research gaps relevant in the current literature, formulating a CR agenda that can guide future CR research. In doing so, it is critical to identify how important the supply chain topic is for strategic decision making when it comes to CR, especially with all its complexity added by hundreds or even thousands of different chain members, resulting in a global network from which reputational damage can arise very quickly via spillover effects, as the Boohoo case shows. To ensure a sustainable supply chain, all stakeholders of a company will increasingly demand information, transparency, and traceability, seeking greater control. In a chain setting, managing these demands is challenging with IT advancements, such as cloud solutions for mitigating risk during global crises, becoming increasingly prevalent. Specifically, companies are moving applications and parts of their IT infrastructure to the cloud to simplify data management to minimize risks, including reputational risks, along the supply chain (Colicchia et al., 2018; Singh, 2021). A traceable and transparent information management system is critical, especially when deliveries of important components for production are delayed or not delivered at all (Colicchia et al., 2018; Golan et al., 2020).

When it comes to information management, transparency and traceability, cyber-attacks can significantly damage and compromise a company’s reputation and, thus, create new risk factors. Similarly, companies along the supply chain can jeopardize CR if they do not perform due diligence or do not comply with the legal regulations that are in place for the enforcement of human rights and sustainability. The primary focus of companies is understandably often on their customers, but given the interlinked nature of CR, they also need to understand their suppliers’ behavior just as well. The pressure on companies to create more transparency regarding the origin of raw materials and the nature of production processes is, therefore, increasingly substantial (Gualandris et al., 2021; Mollenkopf et al., 2022; Roy, 2021).

To capture and manage CR, all stakeholders of a corporate environment should be considered. Looking at the CR concept holistically, it is only possible to manage it along the entire supply chain with all parties actively engaged. The bibliometric mapping shows the multiple fields that connect with CR. It remains, therefore, challenging to capture or explain every detail about the meaning of reputation in a single model. This is particularly important in the supply chain setting, as the Covid-19 pandemic brought to light (Gereffi et al., 2022; Panwar et al., 2022; Phillips et al., 2022), resulting in interdependencies and associated risks of being dependent, when looking at global chains (Alexander et al., 2022; Sauer et al., 2022; Seuring et al., 2022).

Theoretical advancements in CR are needed to offer recommendations for responding to the changing conditions. Those maintaining their CR in the long term will have to do more than ‘communication’ in the future. It will take a great deal of effort, especially in the changing demands on supply chain issues, that requires that all CR dimensions are utilized (see Fig. 3b).

The figure shows the fourth cycle of the scientific path, and it is, thus, a continuation of the first three modes of thinking, indicated in Fig. 3a. Overall, the school of thoughts have the stakeholder approach in common, which is the unifying theoretical foundation over the course of time. This becomes particularly noticeable, since the 2000s. Researchers agree that the topic of CR must be covered from a broader stakeholder perspective, which renders a single-dimensional approach insufficient. It is necessary to go a step further and consider a holistic assessment of CR, including not only financial aspects, but also environmental, ethical, social, cultural, legal, and technical dimensions, which we tried to achieve by identifying and exploring the different dimensions of CR (Baldarelli & Gigli, 2014; De Castro et al., 2006; Singh & Misra, 2021). These are currently manifested in the literature and allow for a forward-looking school that consolidates the insights made thus far.

### Recommendations for Future CR Research in the Supply Chain Context and Beyond

The research questions listed in Table 4 were extracted from the pool of SLR articles dating from 2018 onwards. In the time span considered, we identified a total of 172 questions for further research. To avoid repetition, we summarized and thematically clustered these into 52 questions. Based on our literature assessment, we added 13 CR questions that are specifically relevant for supply chains, resulting in 65 research questions that await empirical treatment to advance theory. On this basis, the implications for further research were assigned to the clusters identified in the bibliometric mapping (right-hand side of Table 4).

**Table 4:** Corporate Reputation: research topics for further scholarly inquiry

Theme/research question	Cluster relevance				Priority rank*
	1	2	3	4	
One: CR impact on supply chains					
1. How does CR develop along the supply chain?		×	×		1
2. How do corporate crises affect industry peers?			×	×	1
3. What is the impact on the supply chain of negative reputation effects resulting from a crisis?			×	×	1
4. What dimensions of CR are relevant for different stakeholders along a supply chain?		×	×	×	1
5. How can CR be utilized to improve supply chain resilience and shield actors from reputational damage?	×		×	×	1
6. What is the relevance of consistency across, and transferability among, different dimensions of CR in supply chains?			×		2
7. What dimensions of CR could spill over from one chain member to another?			×	×	2
8. What are the triggers of CR spillover effects?	×	×	×	×	2
9. How can CR lessons be shared within the supply chain so that all actors benefit from chain e×periences?			×		2
10. Is it possible that one firm’s CR can overrule that of another in the same supply chain?		×	×		3
11. In what ways are the dimensions of CR different for the B2B sector compared to a B2C environment?		×	×		3
12. How far do CR dimensions spill in the supply chain setting?		×	×		3
13. What is the magnitude of CR spillovers on corporations (negative/damage versus positive)?				×	3
Two: CR and value co-creation					
1 How does CR influence the process of value co-creation?		×			1
2. What are the potential limitations of CR in the process of value creation?		×			2
3. What is the precise role of CR in signaling superior value to the stakeholder environment?	×	×			3
Three: Reputational risk and crisis management					
4. How long does it take for a firm’s reputation to recover after a crisis?	×				2
5. Under what conditions and how do organizations respond (or not) to reputational crises?		×		×	2
6. How can CR influence the severity of a crisis?	×				2
7. How do firms repair their CR after a crisis?	×				2
8. What are the reputational consequences of a damaged CR on the perceived market offerings of firms?					2
9. Can social responsibility tarnish a firm’s reputation in a time of crisis?	×				2
10. How do CR and responsibility attribution affect firm value at the onset of a crisis?	×			×	3
11. How can it be ensured that critical voices are heard, and strategies implemented efficiently and effectively to manage reputational risk by a firm?		×		×	3
12. How do disclosures and transparency influence stakeholders’ perceptions of firm reputation?	×			×	3
13. How does the choice of communication language in a crisis influence CR?		×	×		3
Four: Stakeholders’ impact on CR					
14. What are the possible mechanisms that underlie reputational change processes?	×	×	×	×	1
15. How can CR be measured from the perspective of various stakeholders?			×	×	2
16. What is the influence of stakeholders on CR?				×	2
17. How can corporations be influenced by intent to purchase a firm’s market offering, invest in the firm, or join the firm as an employee?		×	×		3
18. What are the indirect and direct impacts of CR on a stakeholder’s behaviors and business outcomes?			×	×	3
19. Which CR KPIs can be measured, in terms of stakeholder behavior, intention or end-states, and traced back to strategic action and stakeholder e×periences and observations?		×	×		3
20. What are different stakeholders’ e×pectations of the inter-relationships between corporate sustainability, image, and reputation?		×	×		3
21. How do different dimensions of CR vary depending on specific audience interests?	×	×	×	×	3
22. How do different stakeholder groups perceive ownership structure, and how do they influence the perception of CR?	×		×	×	3
Five: CR and corporate governance					
23. What are short-term/long-term effects of CR regarding its distinctive self-regulatory focus?			×		3
24. What influence does CR have on the organization’s overall short vs. long-term interests, internal vs. external change processes, company-oriented vs. stakeholder-oriented approaches and organizational vs. societal benefits?	×		×		3
25. What influence do the size, age, and organizational structure of a corporation have on its reputation?	×		×		3
26. What are the effects of specific institutional characteristics of the firm’s home country, such as political, social, and regulatory structures, cultural values, technological development and attitudes toward the natural environment, have on CR?	×	×	×		3
27. How do different organizational structures affect the formation of CR?	×		×		3
28. Do corporate ownership structures influence CR?			×	×	3
Six: Influence of multi-cultural contexts and country of origin effects					
29. What is the importance of national and international differences in stakeholder environmental contexts, and of multi-cultural differences among the stakeholders in CR formation?			×	×	3
30. How does CR work in Middle Eastern and Asian markets?	×		×		3
31. What influence do the industry context, or the country of origin have on CR?		×			3
32. How much do national, regional and industry differences influence CR?		×	×		3
33. How does CR work in emerging markets?	×	×			3
34. What differences exist between CR in western countries compared to emerging markets?		×			3
35. Which cultural dimensions influence the perception of CR?			×	×	3
36. What is the link between a B2B company’s reputation and sustainability marketing in both the developed world and emerging markets?		×			3
Seven: CR and social media					
37. What is the influence of social media platforms on the formation and evolution of CR?			×		3
38. What are the relationships between social media presence, online public opinion and CR?		×	×		3
39. What processes are in play in the formation of CR during an age of advancing digitalization?		×	×		3
40. What are the relevant media influencing CR?		×	×		3
41. What specific options exist for companies to find a hearing and acceptance on online platforms?		×			3
Eight: CR impact on consumer behavior					
42. How does CR shape a customer’s relationship to a company?		×	×		2
43. Does CR influence customers’ cross-buying intention?		×	×		3
44. Is there a relationship between CR and customer loyalty?		×	×		3
Nine: Legal and public affairs					
45. How do changing legal regulations affect a firm’s CR?	×				2
46. What influence do publics have on CR as the most widely defined stakeholder group?		×			2
47. How do the costs and benefits of bribery impact CR?	×				3
48. What are the reputational penalties when outside monitors are appointed at the time of a bribery settlement?	×				3
Ten: Impact of CR on financial performance					
49. What is the impact of CR on the growth of a company or on financial performance?		×		×	2
50. What are the long-term, negative effects on CR of sales losses and a substantial loss of stock market value?		×			2
51. How do company sales, risk profile, and financial performance influence the development of CR?		×		×	2
52. To what e×tent are perceptions of CR driven by non-financial aspects?		×			2

*Rank of research topics: 1 = critical/immediate; 2 = very important/short-term; 3 = important/mid-term

We divide the research questions into ten different themes, indicating distinct research directions. The supply chain section, for example, focuses on how CR originates and develops along the chain and, thus, affects the reputation of individual chain members (Manello & Calabrese, 2019). In addition, questions arise as to what extent reputational effects result from a crisis in the supply chain and how the CR of other chain members could be affected (Lemke & Petersen, 2018; Tannous & Yoon, 2018).

Quite visibly, yet surprisingly, CR academic research in a supply chain context is noticeably underrepresented, and specific CR questions in this area are listed in Table 4 (highlighted in gray). Furthermore, we continued with the ranking of questions beginning with the ones that are currently critical to move the field forward and others that are suitable for subsequent exploration. We encourage future researchers to adopt a supply chain perspective in their CR investigations. The chain setting adds complexity, but it is important to recognize the impact that this research stream can make on supply chain theory development and practice.

Researchers from *Cluster 1*—*Organizational Perspectives* note the possibility that the reputation of one company can override that of another (Burke et al., 2018; Cooper et al., 2018; Park et al., 2020). This work recognizes the importance of understanding how to prevent the transfer of a negative reputation during a crisis. In a similar vein, it is also interesting to learn how to make use of a positive reputation of one supply chain partner to add reputational value to others. This requires further study of spillover effects (Lemke & Petersen, 2018). While it is recognized that CR spills over from one actor to another, it is not known how this occurs in practice. Some CR dimensions may spill directly, while others can spill in an indirect fashion. Some may not spill at all, as they are heavily tied to a single actor (Petersen & Lemke, 2015). Some may spill immediately, while others spill much more slowly. For future research, this raises the questions of which CR dimensions (e.g., innovativeness, working environment, etc.) spill, how far they spill, and what determines the magnitude of the spill. It would be fruitful to explore also which dimensions have the tendency to re-spill from one actor to another and, subsequently, to other actors—like skimming stones on a lake’s surface. Finally, the effect of reputation spills on actors in other supply chains and associated networks is another promising avenue for research.

Within this cluster, organizational authenticity and its influence on corporate purpose as well as CR has been a key area in recent research. One strand of literature seeks to understand the future of work and its influence on organizations (Jiang et al., 2022; Valdés et al., 2022). According to this line of argumentation, the working environment impacts on the overall attractiveness of a firm and, thus, influences its CR. Adding to this, the influence of social regulation (especially CSR) and its regulatory effect on organizations is regarded as an important area for future research. Specifically, work is warranted regarding how CSR leads to spillovers of reputational risks between chain members and ultimately influences stakeholders’ perceptions. This research cluster also identifies a need to address shortcomings in our understanding of how transformative technologies—such as social media—influence the process of reputational spillovers and reputational damage (Nardella et al., 2022). Similarly, the complex role of the state in the formation and evolution of CR is similarly regarded as insufficiently researched. Thus, insights into regulatory efficacy, as well as alternative social regulatory mechanisms effectively shaping CR in the organizational context are called for.

Researchers from *Cluster 2*—*Empirical Perspective* emphasize that the development of reputation in the supply chain warrants further investigation (Karamchandani et al., 2021; Mani & Gunasekaran, 2021; Nurchayati et al., 2020). Specifically, questions arise regarding the impact of crises on CR and on the supply chain (Coombs & Laufer, 2018; Gomez-Trujillo et al., 2020). In this context, there are also considerations in how far reputational crises affect business partners. Extant research stemming from *Cluster 2* recognizes that CR is transmitted throughout a supply chain. However, how such a transfer works and what dimensions of reputation can be transferred remains unclear (Cole & Aitken, 2020; Dhingra & Krishnan, 2021; Wang & Franke, 2020). Hence, research on reputational owners and reputational borrowers is recommended (Petersen & Lemke, 2015).

Current research from this cluster deals mostly with quantifying the relationship between CSR and CR. It calls for more longitudinal, in-depth assessments (McWilliams et al., 2019). A broader range of methods, including ethnomethodological ones and experiments are needed to provide better evidence of causality, and overcome the limitations of cross-sectional survey-based research. Regarding topic focus, this cluster does not pay particular attention to the supply chain context but perceives a need for greater research regarding how to defend and enhance CR in a digital environment (Ertz et al., 2022; Syed Alwi et al., 2020). This involves testing whether digitization is inevitably accompanied by greater customer integration, making the customer an even more integral part of the formation of CR (Morgeson III et al., 2020; Schaarschmidt et al., 2021). For instance, in a social media environment, do company’s customers become more visible, so that its CR becomes more dependent on how others perceive their customers?

Research from *Cluster 3*—*Individualistic Perspective* considers the impact of reputational crises on a company’s market offering and the value co-creation process. Here, researchers call for further attention to be paid to risk management strategies (Arora et al., 2021; Dhingra & Krishnan, 2020; Pérez-Cornejo et al., 2019). Specifically, this should involve preventing reputation loss and restoring lost reputation in a context where supply chains are becoming increasingly complex, globalized, and highly digitalized (Lemke & Petersen, 2018; Quintana-García et al., 2021; Rajagopal et al., 2021; Walsh et al., 2015). Communication styles, company actions, and strategies in such a context also warrant further research (Ajayi & Mmutle, 2021; Busse et al., 2017; Ingenhoff et al., 2018; Singh & Misra, 2021). A blind spot in the assessed literature is the lack of studies considering CR in a multinational context, along truly global supply chains (Abugre & Anlesinya, 2020; Aguilera-Caracuel et al., 2017; Swoboda & Hirschmann, 2017). An accurate examination of the influence of cultural dimensions on the generation and transfer of CR is warranted (Swoboda & Hirschmann, 2017). For instance, the effects of cultural dimensions could be conceptualized and measured based on cultural dimension theory and cultural context theory (Hofstede, 1980).

Recent research within *Cluster 3* focuses on the impact of corporate marketing on corporate brand orientation, corporate brand image, and corporate brand reputation as well as on organization’s stakeholders (Balmer & Podnar, 2021; Melewar et al., 2021). This assesses the degree to which, and how best, a corporation can control its image. Part of this research agenda addresses the importance of integration of communications across a corporation (Chun et al., 2019) and its supply chain. It identifies that further research regarding the influence of departmental reputation or a single employee’s actions on overall CR is warranted (Brown et al., 2022). For instance, if an employee commits a crime or behaves antisocially, what is the effect on CR? Consequently, the relationships between CR and stakeholders’ individual reputations should be investigated further. Finally, research on the role of social media on an individual’s perception of a corporation’s reputation remains limited and the potential mechanisms explaining such relationships are poorly understood (Rutter et al., 2021).

Authors from *Cluster 4*—*Conceptual Perspectives* rely on financial data. The impact of CR on financial performance is often examined in terms of sales and stock market prices (Fasaei et al., 2018; Fombrun et al., 2015; Love & Kraatz, 2017; Zhelyazkov & Gulati, 2016). Moreover, the influence of CR on risk management has increased in importance in the academic literature (del Brío & Lizarzaburu, 2017; Eckert, 2017), especially in the aftermath of the 2007/08 financial crisis (Fourati & Dammak, 2021; Gangi et al., 2020; Sethuraman, 2018; Shim & Yang, 2016; Thakor, 2015).

Recent research from authors within *Cluster 4* seeks to understand the effect of CR on buyers’ intentions. They identify that CR is of special importance in an e-commerce environment because of the typically trust boosting effects of face-to-face encounters, which are absent in an online setting (Fombrun et al., 2022). Consequently, in a digital environment, other strategies for augmenting CR should be identified (Fombrun et al., 2020). In response, many researchers focus on the social and ethical values of the corporation (Bundy et al., 2022). As an outcome, researchers call for further research on the topic of responsible leadership and CR, including the impact of social and ecological responsibility on stakeholder perceptions (Freeman & Auster, 2021). This dovetails with a need for work on how stakeholders’ judge the sincerity of a corporation’s social and ethical pronouncements.

Stakeholder theory remains at the center of *Cluster 4’s* research, which continues to address the influence of stakeholders on the overall reputation of a company (Baah et al., 2021; Barnett & Leih, 2018; Fombrun et al., 2015; Ghadge et al., 2020; Waldner & Willems, 2020). From this perspective, future research should investigate the level of influence individual stakeholder groups have on a company’s reputation. Furthermore, the question of suitable methods and metrics for CR remains a key consideration. It is recommended to continue researching the composite elements of CR to determine how stakeholders and supply chain partners affect the company’s reputation (Baah et al., 2020, 2021; Fombrun et al., 2015; Walsh et al., 2015). Stakeholder-based perspectives should recognize the growing importance of online and social media environments. Specifically, studies should seek to understand social media’s role in the context of CR formation (Dijkmans et al., 2015; Hartmann, 2021; Ott & Theunissen, 2015; Waldner & Willems, 2020; Zheng et al., 2018). To date, the literature on this remains nascent with only a few articles directly considering the influence of digital media on the development of CR (Mingione & Abratt, 2020; Schaarschmidt & Walsh, 2020). Key questions for further research include how communication channels affect the nature of information exchange between stakeholders (Gomez-Trujillo et al., 2020; Quintana-García et al., 2021; Syed Alwi et al., 2020) and how the nature of the media affects the degree to which CR is transferred from one supply chain partner to another (Azadegan et al., 2020; Hartmann, 2021; Mihardjo et al., 2020).

The four clusters show substantial room for further exploration. However, none of the clusters directly addresses sustainability aspects. In the SLR, we recognize the lack of attention placed on topics such as green, responsible, and sustainable supply chains, when it comes to the CR debate. This is a vital area for exploration. Consequently, future research should understand the effects of CR on corporations’ sustainability actions and their responsiveness to societal developments. This could include an assessment of how CR activities differ in international versus national supply chains and how CR affects assessments of whether a particular supply chain is regarded as sustainable or not.

### Practical Implications

In the SLR, the impact that others have on CR is particularly noticeable in the supply chain context. Managers should understand and use reputational mechanisms to their advantage. Either their company has built up a certain reputation and can spread reputational dimensions to others or they are in the position of the reputational borrower, that benefits from or is damaged by reputational triggers (e.g., offer, communication, action) of others (Lemke & Petersen, 2018). Managers also must pay attention to the ‘ones that care.’ These stakeholders are reputational reflectors (e.g., customers), whose awareness and relevance cause spillovers to occur. Relationships with these stakeholders should be managed well, so that spillovers can be controlled to a greater extent.

Companies can no longer manage supply chains like in the pre-COVID-19 era. Transparency, sustainability, and security of supply are essential for mitigating reputational risks along the supply chain (Gereffi et al., 2022; Phillips et al., 2022; Seuring et al., 2022). Transparency must also exist when it comes to information flows, as clear and direct communication, as a reputational trigger, is a fundamental part of reputation management within supply chains (Lemke & Petersen, 2013; Panwar et al., 2022).

Finally, managers should carefully consider the importance of sustainability criteria and social standards as part of CR because modern customers are increasingly critical and less forgiving (Yang et al., 2021). SCM is currently troubled by a lack of visibility throughout extended supply chains, as corporations often have complex supplier networks operating at multiple tiers (Panwar et al., 2022). Consequently, to minimize reputational risks, it could be useful integrating advanced information technologies to significantly improve visibility and, thereby, become more responsive to major disruption and variability within supply chains (Phillips et al., 2022; Sauer et al., 2022).

## Limitations

While this paper provides a research agenda for future CR topics, based on a SLR, we acknowledge that this study has several limitations. We explored the topic of CR from a business perspective which might be a limitation of this paper. Aguinis et al. (2022) recently suggested to integrate more practitioner insights into academic research, which is also supported by other scholars (Schön, 1995; Stokes, 1997; Thompson & Thompson, 2008). With respect to our review, we excluded non-peer-reviewed publications such as books, conference papers, white and gray literature as well as non-English publications. Including papers published in only ABS 2 to 4* ranked journals also limited the scope but maintained a focus on the research frontier. We did not specifically capture the broader societal themes (macro) that are relevant, regarding political, technological, environmental, and economic global debates. In our SLR, we identified the most popular theories applied in the four research clusters. We did not capture how studies relate to each other and the methods they used for their investigations in great depth. More fine-grained work understanding the dynamics of each cluster is warranted.

## Conclusion

CR is an important concept, affecting value creation and destruction along supply chains. Whereas early work on value co-creation focused on seller-customer dyads, this article introduces and advocates a supply chain perspective. This recognizes the potential for reputational spillover effects in a supply chain, as witnessed in the case of Boohoo.com (Levitt, 2020), and recently proposed legislative changes that widen the remit of due diligence to include supply chain partners (Australian Government, 2018; European Parliament, 2021; UK Parliament, 2015). Consequently, CR should be studied within a holistic SCM context. However, as demonstrated by the SLR, a supply chain perspective is typically lacking within the CR literature while the supply chain literature falls short on its treatment of CR.

In addition to CR, we acknowledge that other intangible assets are strongly relevant in a supply chain context too, such as relational capital, collaboration skills, and network capabilities, among others. It is important to differentiate intangible assets in a supply chain context, study their connections as well as their effects on the supply chain. However, we firstly need to provide foundational research on CR before investigating the interplay between different intangible assets in a SCM context. This paper, thus, represents a starting point for further research on CR and its connection with SCM and potential reputational risks. The latter includes reputational spillovers. It is an attempt to rectify an existing bias and provide a basis for future studies in this vital area. For this purpose, the SLR allows us to define CR more comprehensively and the subsequent bibliometric mapping provides strategic research directions that are rooted in four literature clusters. Based on the analysis, we identify and map out future directions for the academic study of CR with a supply chain focus, linked to recent articles in each of the four CR research clusters. We hope that our assessment will motivate researchers to consider how CR is created, maintained, and destroyed in a wider supply chain context.
